# Radon exhalation rate and natural radioactivity in the building materials used in metropolitan Jakarta and its surrounding areas, Indonesia

**DOI:** 10.3389/fpubh.2025.1539957

**Published:** 2025-02-18

**Authors:** Eka Djatnika Nugraha, Oumar Bobbo Modibo, Wahyudi, Radhia Pradana, Rima Agustin Merdekawati, Kartini Megagasri, Abdussalam Topandi, Agus Nur Rachman, Rusbani Kurniawan, Evans Azka Fajrianshah, Nurahmah Hidayati, Ilma Dwi Winarni, Ilsa Rosianna, Leons Rixson, Dikdik Sidik Purnama, Heru Prasetio, Shinji Tokonami

**Affiliations:** ^1^Research Center for Safety, Metrology, and Nuclear Quality Technology, Research Organization for Nu-clear Energy (ORTN), National Research and Innovation Agency (BRIN), South Tangerang, Indonesia; ^2^Department of Radiation Science, Graduate School of Health Sciences, Hirosaki University, Hirosaki, Japan; ^3^Directorate of Competency Development, The National Research and Innovation Agency of Indonesia (BRIN), Jakarta, Indonesia; ^4^Polytechnic of Nuclear Technology, The National Research and Innovation Agency of Indonesia (BRIN), Sleman, Indonesia; ^5^Polymer Chemical Engineering, Polytechnic STMI of Jakarta, Jakarta, Indonesia; ^6^Research Center for Nuclear Fuel Cycle and Radioactive Waste Technology, Research Organization for Nuclear Energy (ORTN), National Research and Innovation Agency (BRIN), South Tangerang, Indonesia; ^7^Research Center for Nuclear Beam Analytics Technology, Research Organization for Nuclear Energy (ORTN), National Research and Innovation Agency (BRIN), South Tangerang, Indonesia; ^8^Institute of Radiation Emergency Medicine, Hirosaki University, Hirosaki, Japan

**Keywords:** building material, natural radioactivity, radon, RESRAD-BUILD, effective dose, exhalation rate

## Abstract

**Introduction:**

Creating a safe living environment involves using healthy and sustainable building materials. Humans are exposed to natural radionuclides, such as ^226^Ra, ^232^Th, and ^40^K decay series, found in building materials that pose a radiological hazard. This study is aimed to investigate the radionuclides content of building materials used in Jakarta and its surrounding areas. The computer code RESRAD-BUILD was used to calculate the annual effective dose received by an adult living in a typical room constructed with the studied building materials.

**Methods:**

Samples such as sand, cement, bricks, and Autoclaved Aerated Concrete (AAC) were collected. The ^222^Rn surface exhalation rate was determined using the closed chamber method using RAD7, while the activity concentration of natural radionuclide was measured using a gamma spectrometer.

**Results and discussion:**

The ^222^Rn surface exhalation rate varies from 4 × 10^−2^ to 1.6 × 10^0^ mBq m^−2^ s^−1^ with an average of 4 × 10^−1^ mBq m^−2^ s^−1^. The average ^222^Rn exhalation rate of the building materials studied was much lower than the global average value of 1.6 × 10^1^ mBq m^−2^ s^−1^. The average activity concentration values of ^232^Th (21 Bq kg^−1^) and ^40^K (217 Bq kg^−1^) in all building materials studied are lower than the global average values of 45 and 412 Bq kg^−1^. In comparison, the average activity concentration of ^226^Ra (34 Bq kg^−1^) is similar to the global average value of 32 Bq kg^−1^. Furthermore, the assessed radiological hazard from the measured building material has an average activity index of 0.3, while the RESRAD-BUILD estimated total annual effective dose for a typical house constructed using a mixture of the building materials was 0.11 mSv, in which indoor ^222^Rn alone represents 92% of the total. From the assessment results, the building materials in Jakarta and its surrounding areas do not pose significant concerns regarding radiological hazards. However, the higher contribution of ^222^Rn suggests the need for a large-scale indoor ^222^Rn survey in the study area.

## Highlights

- Transforming living spaces into safe environments that prioritize human health, environmental protection, and sustainable development is among the goals adopted by the United Nations under the framework of the Sustainable Development Goals (SDGs).
- The quality of building materials plays a vital role in the effort to create better cities. Building materials, such as bricks, sand, gravel, or Autoclaved Aerated Concrete (AAC) contain natural radioactive elements.
- This requires monitoring and assessing the risks of radiological exposure these materials would pose to the public within these infrastructures.
- This study monitored the concentration of natural radioactivity of the natural radionuclides, such as ^226^Ra, ^232^Th, ^40^K, and ^222^Rn, and the surface exhalation rate in building materials used in one of the largest urban concentration areas in the world—the megalopolis of Jakarta in Indonesia. A total of 81 building material samples were collected from 17 regional areas for this purpose.

## 1 Introduction

To transform the world into a better society, the United Nations adopted 17 Sustainable Development Goals (SDGs) to be achieved by 2030. These SDGs foster the development of a more prosperous, healthy, safe, and peaceful living environment. Building materials contribute directly to achieving SDGs goals, especially SDGs goals 3, 7, 9, 11, 12, 13, and 15 (1). The use of non-hazardous building materials was one of the key aspects of green building to enhance human health (2).The use of non-hazardous building materials was one of the key aspects of green building to enhance human health. This poses a major challenge, particularly for megacities in developing countries. The safety of building materials counts among others, especially when it comes to their natural radioactivity content. Human exposure to radiation from natural radionuclides is the primary source of the total annual effective dose received from all sources—both natural and artificial (3). Among the natural sources, the level of exposure varies depending on the exposure pathway. On average, each individual receives an annual radiation effective dose of 2.4 mSv, consisting of 1.1 mSv from inhalation of ^222^Rn gas and 0.48 mSv from external gamma radiation, representing 48 and 20% of the total effective dose received annually, respectively. The remaining amounts are 0.29 mSv from ingestion (12%), and 0.39 mSv from cosmic radiation (16%) (4). ^222^Rn is the main contributor to the annual effective dose. ^222^Rn is a radioactive noble gas belonging to the ^238^U decay series found in soil and rocks (5). Epidemiological studies and dosimetric modeling provide evidence of a direct link between exposure to radon (^222^Rn) and the risk of lung cancer (6–10). ^222^Rn is recognized as a carcinogen element by the International Agency for Research on Cancer (IARC), and according to the World Health Organization (WHO), ^222^Rn is the second leading cause of lung cancer after smoking (11, 12). In addition, in certain countries, studies have been carried out to determine lung cancers attributable to exposure to ^222^Rn (13, 14). Furthermore, external gamma radiation is the second contributor to the annual effective dose after ^222^Rn (4). ^226^Ra, ^232^Th, and ^40^K are primordial radionuclides in soil and rocks. Their decay products are gamma emitters for ^226^Ra and ^232^Th and a direct gamma emitter for ^40^K, which participate in the external gamma exposure of humans (15). In some areas of the world, the building materials used to build houses/offices mostly come from rocks and soil, which contain natural radionuclides such as the ^238^U and ^232^Th decay series as well as ^40^K (16, 17). These building materials often constitute a significant source of indoor ^222^Rn and external gamma exposure.

In urban areas, the majority of houses use walls as the primary building material, made of red brick or concrete blocks and usually coated with cement plaster (18). The main ingredient in cement and red brick is clay, which consists of silicate, aluminum oxide, and limestone, with calcium carbonate being the most significant compound. In big cities, such as Jakarta, autoclaved aerated concrete (AAC) is widely used for construction. AAC is made from quartz sand, cement, and developer. Also, sand material is widely used as a mixture for cement, concrete, or bricks (19). Natural radionuclides in building materials can pose external and internal radiation hazards to building occupants. The external radiation hazards are attributable to gamma radiation from the decay of radionuclides in the material (^226^Ra, ^232^Th, and ^40^K), and the internal radiation hazards are, in reality, due to the inhalation of radionuclide ^222^Rn and ^220^Rn decay products (20–22). Few researchers have conducted analyses of natural radionuclide content in building materials in the South East Asia region. In Malaysia, Abdullahi et al. analyzed tile materials, red bricks, cement bricks, sand, cement, gravel, white cement, fly ash, feldspar, lime, kaolin, pottery, clay soil, glaze, and talc with the results of the overall average activity concentration of all building materials ranging from 9.6 ± 0.7 to 222.8 ± 5.1 Bq kg^−1^; 8.6 ± 1 to 274.4 ± 8.1 Bq kg^−1^; and 46.3 ± 6.5 to 1589.2 ± 21.1 Bq kg^−1^ for ^226^Ra, ^232^Th, and ^40^K, respectively (23). Similar research has also been carried out on cement, gypsum, and sand materials, with the results of calculations obtaining the highest activity concentration values of ^226^Ra, ^232^Th, and ^40^K found in sand samples at 42.12, 27.79, and 316.2 Bq kg^−1^, respectively (24). Knowing radionuclide concentrations in building materials is vital in estimating potential radiological hazards for building occupants because most people spend 80% of their time indoors (25). This research will measure the activity concentrations of ^226^Ra, ^232^Th, ^40^K, and ^222^Rn, as well as the exhalation rate of ^222^Rn gas in building materials such as sand, cement, bricks, and AAC from Jakarta and its surrounding areas.

Recently, the Indonesian government has been actively promoting the usage of environmentally friendly materials (eco-materials) in several areas, particularly in the construction sector, to achieve sustainable development goals, create a better environment, and provide affordable housing for low-income communities (26). An overall assessment of the safety of these materials is essential for a sustainable environment, including radiological hazards often associated with building materials (27, 28).

Until now, no investigation has been conducted on the local building materials to determine radionuclides concentrations and the ^222^Rn surface exhalation rate used in Jakarta and its surrounding areas. This study aims to undertake an extensive sampling of building materials in Jakarta and the surrounding areas, the major city of Indonesia, followed by laboratory analysis of ^226^Ra, ^232^Th, ^40^K, and ^222^Rn surface exhalation rate calculations from these samples that are also used as building materials. To determine the effective dose received by an adult from the exposition to the radionuclides contained in the building materials, the RESRAD-BUILD computer code was used to analyze the contributions of various exposure pathways.

## 2 Materials and methods

### 2.1 Study area

**Figure 1:**
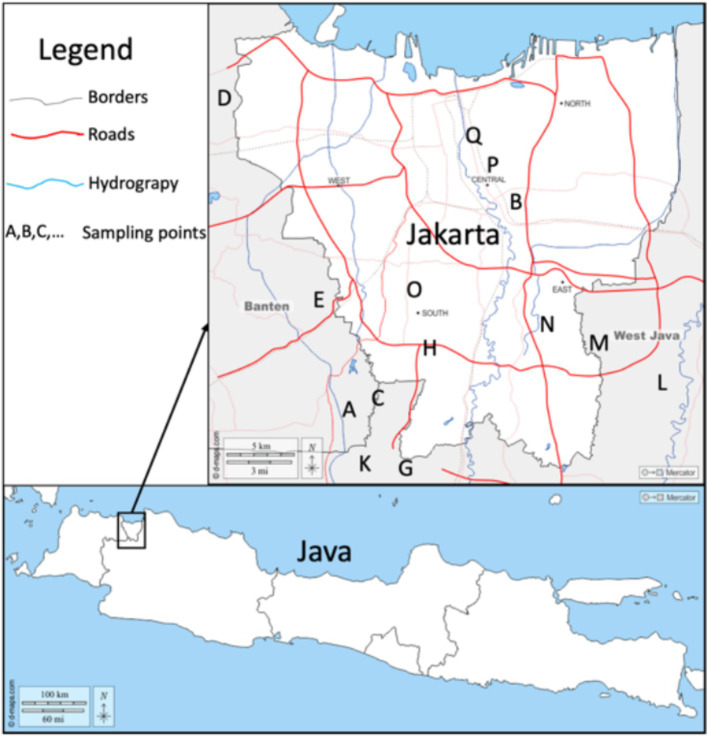
Map of the sampling points in the study area. Reprinted with permission © 2007–2025 https://d-maps.com; data from https://d-maps.com/carte.php?num_car=135659&lang=en and https://d-maps.com/carte.php?num_car=133900&lang=en were combined to create the figure.

The study area in this study is Jakarta—a megacity of Indonesia and its surroundings located on Java Island (Figure 1). Jakarta city and the surrounding areas were chosen as the research locus because it has a reasonably high population with high levels of human activity indoors, both in the office and at home. Based on the Office of Statistical Agency (BPS) data for 2022, the population of Jakarta, West Java, and Banten provinces are 10,680,000, 49,405,000, and 12,252,000 people, respectively (18). Java Island is home to 154,000,000 people, 56% of the country’s total population of 275,000,000 people, making Java the most populated island in the world. Jakarta and its suburbs are home to almost a quarter of Java’s population (18). Jakarta is a tropical, humid city with annual temperatures between 24 and 34°C and a relative humidity of 75–85%. The average mean temperatures are 26°C in January and 28°C in October. The annual rainfall is more than 1,700 mm. Sea winds often modify temperatures. Jakarta, like any other large city, also has its share of air and noise pollution (29).

### 2.2 Sampling and preparation of building materials

A total of 81 samples of building materials are used in the study area, among the types and brands, with details of 19 sand samples, 16 cement samples, 13 brick samples, 17 AAC samples of 10 cm thick, and 16 AAC samples of 7 cm thick. Sampling was carried out randomly at 17 regional areas, namely, Pondok Cabe (A), Pisangan (B), Cirendeu (C), Soekarno Hatta Airport (D), Serpong (E), Balaraja (F), Cikupa (G), Lebak Bulus (H), Cimanggu (I), Parung (J), Sawangan (K), Jatiasih (L), Jatiwarna (M), Bambu Apus (N), Kebayoran Lama (O), Salemba (P), and Kemayoran (Q). Details of building material samples are shown in Table 1. Samples were prepared in the laboratory using a similar protocol to Ndjana Nkoulou et al. study (16). First, they were dried using an oven set at 105°C for 24 h. After that, the samples were weighed, and their volumes were determined. Volume measurements were carried out directly on the material for brick and AAC samples. In contrast, volume measurements were carried out for sand and cement samples by measuring the volume of the sample holder.

**Table 1:** The details of sampling area and building material type.

Area code	Sampling area	Sample code (material type)
A	Pondok Cabe	A1-2 (Sand); A3 (Brick); A4 (7 cm AAC); A5 (10 cm AAC)
B	Pisangan	B1 (Sand); B2 (Cement); B3 (Brick); B4 (7 cm AAC); B5 (10 cm AAC)
C	Cirendeu	C1-2 (Sand); C3 (Cement); C4 (Brick); C5 (7 cm AAC); C6 (10 cm AAC)
D	Soekarno Hatta Airport	D1 (Sand); D2 (Cement); D3 (Brick); D4 (7 cm AAC); D5 (10 cm AAC)
E	Serpong	E1 (Sand); E2 (Cement); E3 (Brick); E4 (7 cm AAC); E5 (10 cm AAC)
F	Balaraja	F1 (Sand); F2 (Cement); F3 (Brick); F4 (10 cm AAC)
G	Cikupa	G1 (Sand); G2 (Cement); G3 (Brick); G4 (7 cm AAC); G5 (10 cm AAC)
H	Lebak Bulus	H1 (Sand); H2 (Cement); H3 (Brick); H4 (7 cm AAC); H5 (10 cm AAC)
I	Cimanggu	I1 (Sand); I2 (Cement); I3 (Brick); I4 (7 cm AAC); I5 (10 cm AAC)
J	Parung	J1 (Sand); J2 (Cement); J3 (Brick); J4 (7 cm AAC); J5 (10 cm AAC)
K	Sawangan	K1 (Sand); K2 (Cement); K3 (Brick); K4 (7 cm AAC); K5 (10 cm AAC)
L	Jatiasih	L1 (Sand); L2 (Cement); L3 (7 cm AAC); L4 (10 cm AAC)
M	Jatiwarna	M1 (Sand); M2 (Cement); M3 (7 cm AAC); M4 (10 cm AAC)
N	Bambu Apus	N1 (Sand); N2 (Cement); N3 (7 cm AAC); N4 (10 cm AAC)
O	Kebayoran Lama	O1 (Sand); O2 (Cement); O3 (Brick); O4 (7 cm AAC); O5 (10 cm AAC)
P	Salemba	P1 (Sand); P2 (Cement); P3 (Brick); P4 (7 cm AAC); P5 (10 cm AAC)
Q	Kemayoran	Q1 (Sand); Q2 (Cement); Q3 (7 cm AAC); Q4 (10 cm AAC)

### 2.3 Determination of the ^222^Rn surface exhalation rates

^222^Rn surface exhalation rate is the flux of ^222^Rn per square meter per second or per hour from the source, such as rocks, soil, or building materials (30, 31). There are several methods for measuring ^222^Rn surface exhalation rate, namely the closed-chamber method (CCM) and the open-chamber method (OCM). The closed-chamber method (CCM) is commonly used to measure the exhalation rate in building materials by putting the sample inside an airtight container and measuring the ^222^Rn activity concentration in the air in the container (32, 33).

**Figure 2:**
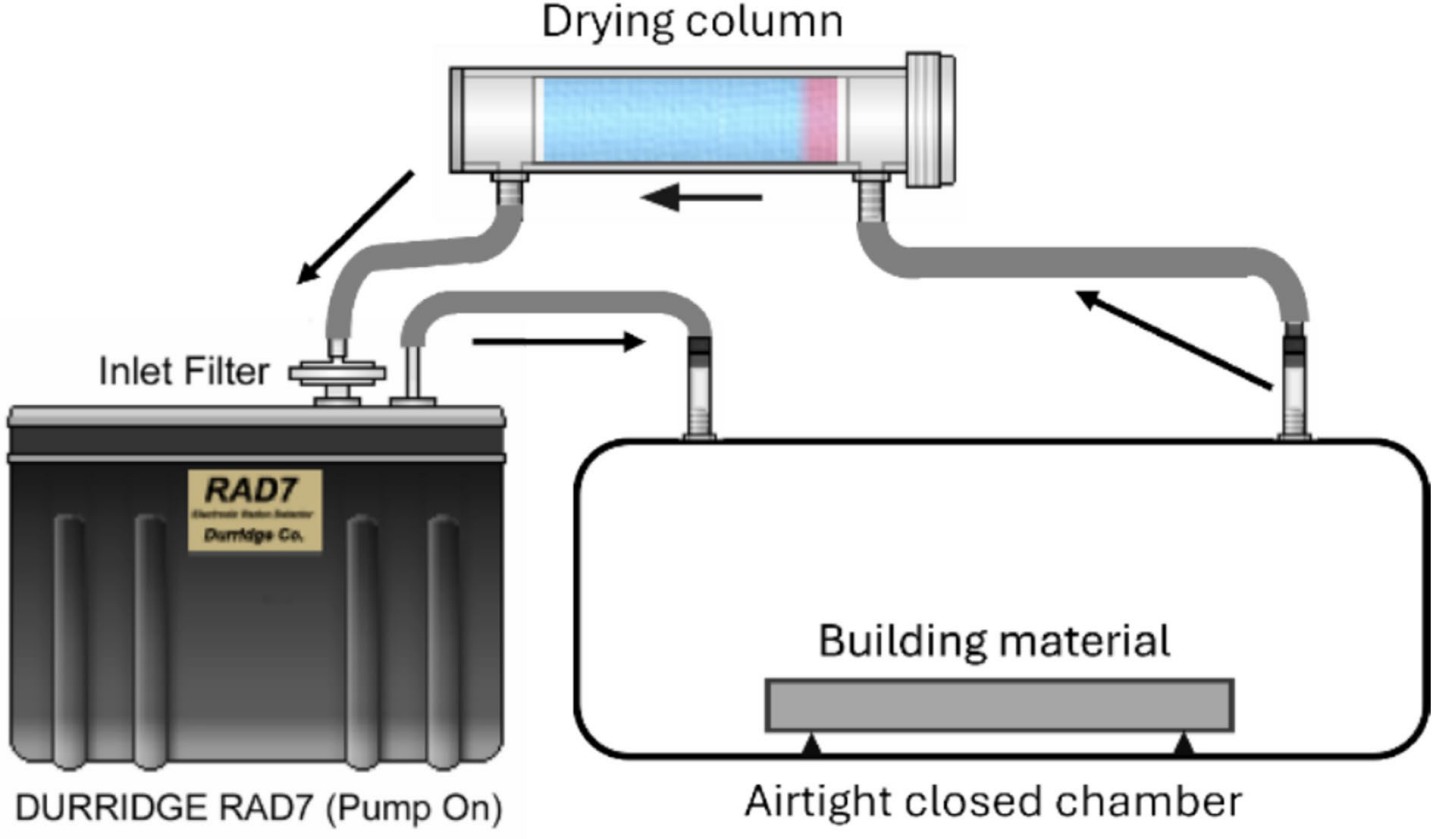
The diagram of ^222^Rn exhalation rate measurement by RAD7.

^222^Rn activity concentration was measured using a RAD7 ^222^Rn monitor (DURRIDGE Company, Inc., USA) with an *α* solid-state detector. The diagram illustrating the measurement of the ^222^Rn exhalation rate by RAD7 is presented in Figure 2. When the container containing the sample is connected to the detector, ^222^Rn gas accumulated due to the exhalation of ^222^Rn in the sample will be pumped to flow into the drying column (Drierite, Drierite Company, Inc., USA) and then into the inlet filter of the RAD7 tool. In RAD7, the sample air will decay so that *α* particles emitted from the polonium isotope will be detected. RAD7 uses α spectrometry techniques to convert α particles into electrical signals. This detector can also separate electrical pulses produced from ^218^Po and ^214^Po with energies of 6 and 7.69 MeV, respectively (34, 35). The sampling cycle was set for 1 h with 6 cycles. The spectrum obtained was then analyzed using Capture software provided by DURRIDGE. ^222^Rn activity concentration measurements were conducted in dry conditions (relative humidity less than 10%). After each measurement, the ^222^Rn inside the RAD7 is purged by pumping clean air. The RAD7 was calibrated for ^222^Rn measurement in the ^222^Rn chamber of IREM (Institute of Radiation Emergency Medicine), Hirosaki University, Japan, which annually conducts intercomparison with the Federal Office for Radiation Protection (BfS), Germany. The ^222^Rn surface exhalation rate is determined using Equation 1 (35).


(1) $$E_{Rn}=\frac{C_{Rn}\times \lambda_{Rn}\times V}{A_{s}\left(1-e^{-\lambda t}\right)}$$

where *E_Rn_* is ^222^Rn surface exhalation rate in Bq m^−2^ s^−1^, *C_Rn_* is the average of the stabilized ^222^Rn activity concentration in Bq m^−3^ obtained by direct measurements using RAD7 device, *V* is the effective volume of the closed chamber as in the chamber volume of 1.42 × 10^−2^ m^3^ decreased by sample volume, *λ_Rn_* is ^222^Rn decay constant in 2.1 × 10^−6^ s^−1^, and *A_s_* is the surface area of the sample in m^2^.

### 2.4 Determination of ^232^Th, ^226^Ra, and ^40^K activity concentrations in the building materials

After the measurements of ^222^Rn surface exhalation rates, the samples were crushed, sieved, and dried in an oven for the second time to remove moisture contained in the materials. After that, the samples were weighed and put into a U8-type vial container, a cylindrical polypropylene container sized 48 mm × 55 mm. The samples in the vials were then tightly sealed to prevent ^222^Rn gas from escaping from inside to allow secular equilibrium to be achieved after 30 days. The measurements are conducted using a calibrated gamma spectrometry system with a *p*-type High Purity Germanium detector (HPGe), GEM60-83-XLB-C-SMP (ORTEC, USA), with a relative efficiency of 60% and a resolution of 1.86 at 1.33 MeV of ^60^Co. The detector is mounted inside a 10-cm thick cylindrical lead shielding, lined with a layer of tin and copper to prevent ambient gamma interference on the measurements, and the X-rays of Compton scattering in the lead reach the detector. The pulse from the detector is analyzed by a multichannel analyzer that is directly connected to a PC with the associated reading software Gamma Vision (ORTEC, USA).

The calibration of the gamma spectroscopy system utilizes a soil matrix reference material from the intercomparison network of Analytical Laboratories for the Measurement of Environmental Radioactivity (ALMERA-IAEA), CRM IAEA soil 448, and CRM IAEA Soil 444. To maintain the reliability and validity of the spectrometry system, the laboratory keeps a routine internal calibration using a mixed radionuclide source. The laboratory also participates annually in the intercomparison network of ALMERA and the Indonesian metrology network for radionuclide measurement.

The ^226^Ra activity concentration is obtained by analyzing the photoelectric peaks of 295 keV; 351 keV of ^214^Pb; and the photoelectric peak of 609 keV of ^214^Bi. ^232^Th activity concentration is obtained by analyzing the photoelectric peaks of 238 keV of ^212^Pb and 911 keV of ^228^Ac. ^40^K activity concentration is determined by examining the single photoelectric peak of 1,460 keV (36). The measurement duration for each sample was 80,000 s. Equation 2 was used to determine the activity concentration *A* (Bq kg^−1^) of each radionuclide (15, 37, 38).


(2) $$A=\frac{C_{s}-C_{bg}}{\varepsilon \cdot \gamma \cdot W\cdot fc}\pm k\cdot U\;$$

where *C_s_* is the count rate of the sample (count s^−1^); *C_bg_* is the count rate of the background (count s^−1^); 𝜀 is the detector efficiency; 𝛾 is the gamma emission probability; *W* is the sample weight (kg); *f*_c_ is the correction factor; *k* is the coverage factor in *k* = 1.96 for a confidence interval of 95%; and *U* is the combined uncertainties of the measurements calculated obtained using Equation 3 (39).


(3) $$U=\sqrt{\sum u^{2}}$$

Where *u* is the relative uncertainty from each sample count standard deviation, detector efficiency, measured sample weight, gamma emission probability, growth factor, attenuation factor, and summing factor. We also analyze the background radiation for each radionuclide to determine the detection limit (LD) using Equation 4 (40).


(4) LD=DT+kU

Where DT is the decision threshold based on the measured background, *k* is the coverage factor, and *U* is the uncertainty in measurement. The ^40^K, ^226^Ra, and ^232^Th detection limits were 0.21, 0.08, and 0.08 Bq kg^−1^, respectively.

### 2.5 Indoor ^222^Rn activity concentration emitted by the building materials

Indoor ^222^Rn concentration originates from sources such as inside/outside air exchange, water supply, cracks in the house’s basements, and building materials used, especially when the house is built from bricks, sand, and rocks. The contribution of building materials to the indoor ^222^Rn concentration is often significant. It is possible to assess that contribution by knowing that ^226^Ra contains the building materials since ^222^Rn is a ^226^Ra decay product. Equation 5 expresses the indoor ^222^Rn from the ^226^Ra in building materials in a steady state condition (16, 41, 42).


(5) $$C_{222_{Rn}}=\frac{C_{226_{Ra}}\times \varepsilon \times \lambda \times \rho \times r\times A}{V\left(\lambda +\tau \right)}\;$$

where $C_{226_{Ra}}$ is the ^226^Ra concentration in the building materials (Bq kg^−1^); ε the emanation fraction (0.2); λ the ^222^Rn decay constant (0.00756 h^−1^); τ the air exchange rate (0.5 h^−1^), ρ and r is the density (100 kg m^−3^) and half-thickness layer (7 cm) of the structural elements (wall and floor) of the room, respectively; and A and V are the room surface area (m^2^) and inner volume (m^−3^), respectively. Activity index I was defined to estimate the radiological risk presented by building materials.

### 2.6 Activity index

The activity index I is calculated using ^226^Ra, ^232^Th, and ^40^K activity concentrations in the building materials. The Equation 6 expresses the different coefficients of calculation (43).


(6) $$I=\frac{C_{226_{Ra}}}{300}+\frac{C_{232_{Th}}}{200}+\frac{C_{40_{K}}}{3000}$$

### 2.7 Annual effective doses from external, inhalation, and ingestion obtained using RESRAD-BUILD computer code

**Figure 3:**
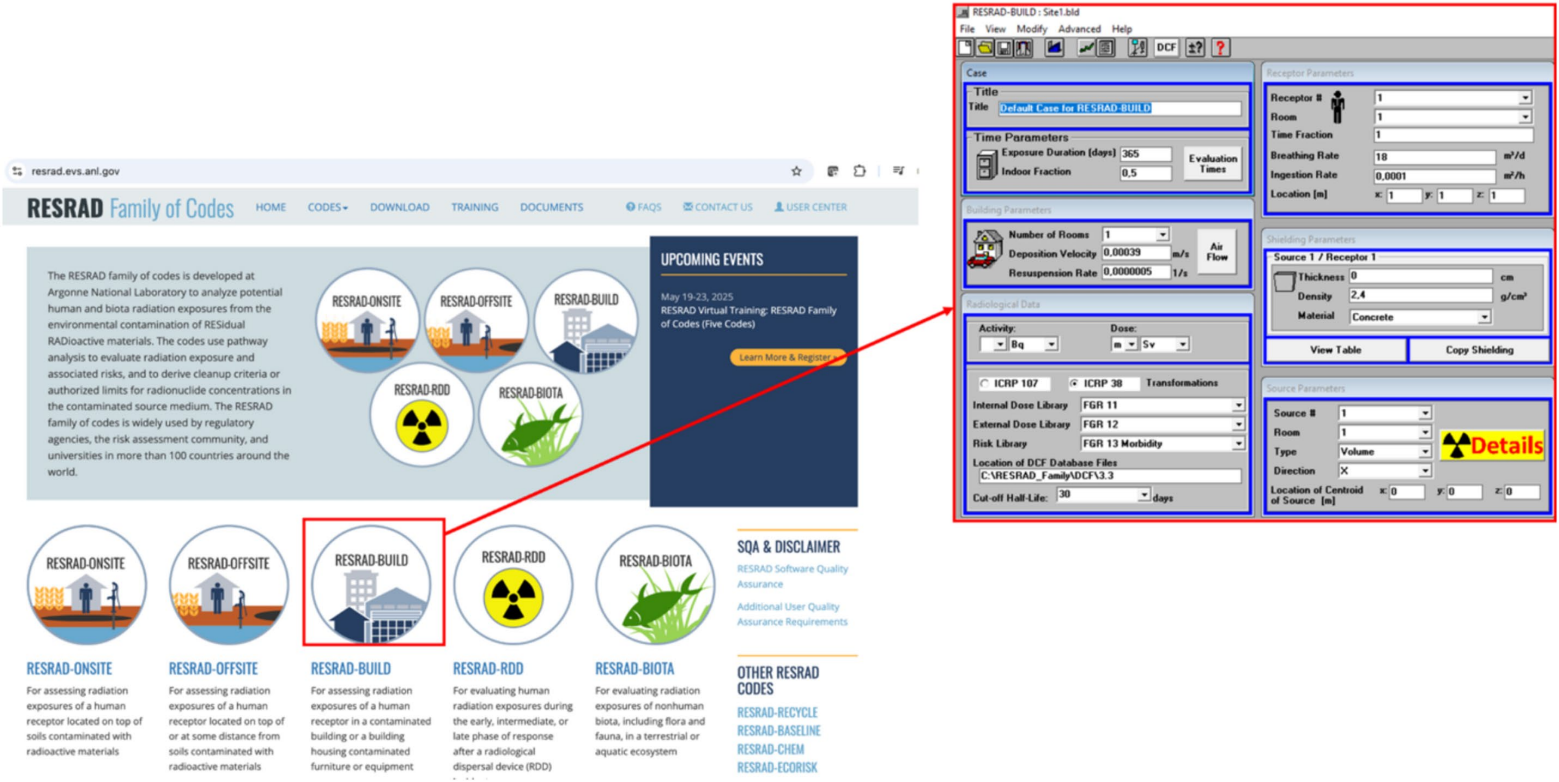
RESRAD web page interface (44). Screenshot (left) from Argonne National Laboratory, RESRAD Family of Codes.

The assessment of the annual effective doses from external, inhalation, and ingestion received by an individual living in a typical house in the study area built using the studied building materials can be obtained using RESRAD-BUILD presented in Figure 3. This computer code is an open-access software developed by the Argonne National Laboratory (Argonne, USA) (41, 44). In detail, the dose resulting from exposure to external radiation emitted by walls and floor, dose received from inhalation of ^222^Rn decay products, and dose from ingestion of radionuclides deposited and resuspended in the house are obtained from RESRAD-BUILD computer software. The exposure scenario is for an average adult who is 1.60 m in height and 80 kg of weight. The flowing parameters were used along with radionuclide concentrations of building materials. The indoor occupancy factor is 0.8 (16, 44). A room model with the dimensions of 4 m × 3 m × 3 m is considered, the half layer walls’ thickness of 7 cm, 1 number of rooms per occupant, radionuclides deposition velocity of 0.01 m s^−1^, radionuclides resuspension rate of 5 × 10^−7^ s^−1^, adult individual breathing rate of 20 m^3^ day^−1^ were used (16, 45, 46).

## 3 Results

### 3.1 ^222^Rn activity concentration and surface exhalation rate of building materials

**Figure 4:**
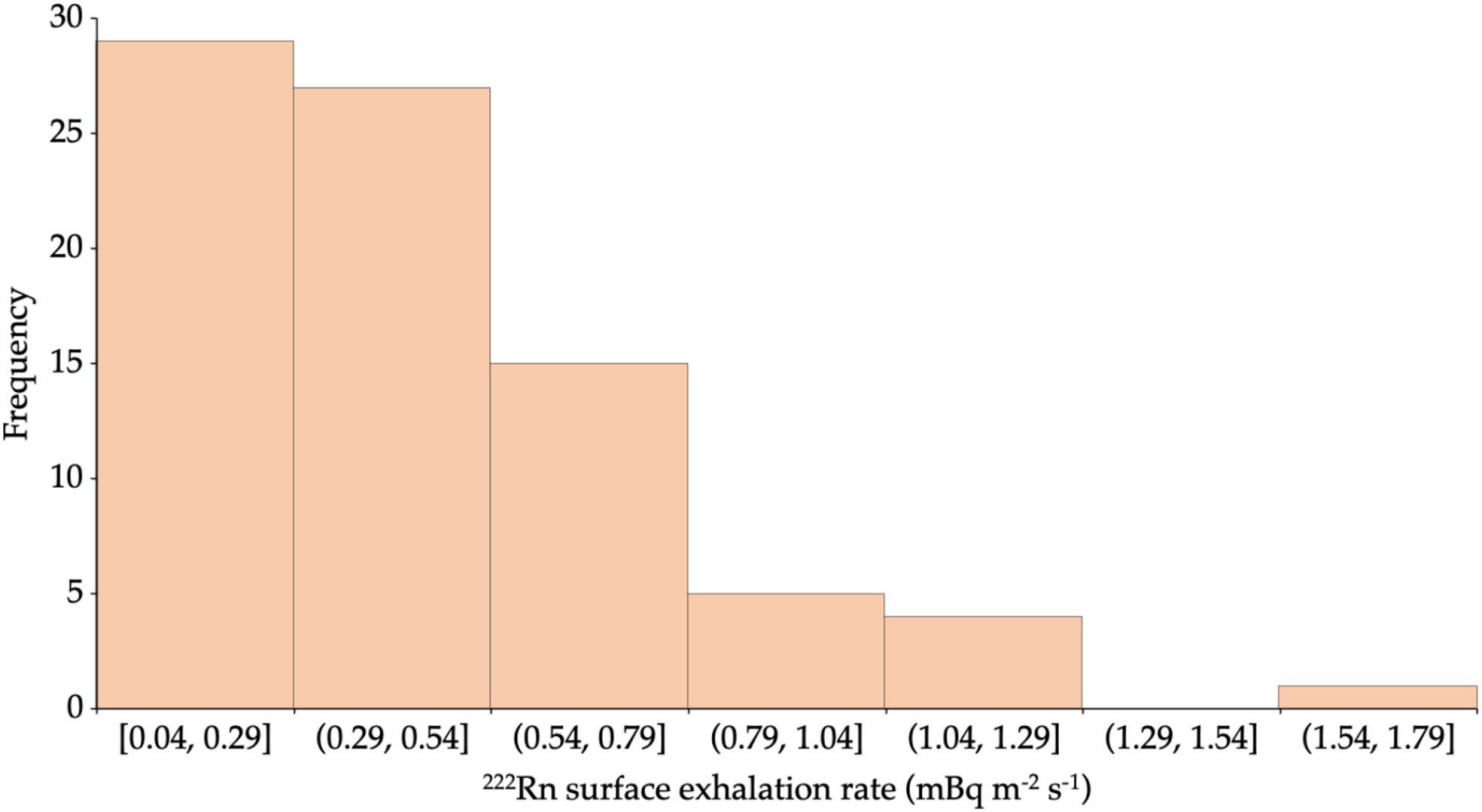
^222^Rn surface exhalation rate distribution from all studied building materials.

The ^222^Rn activity concentration values measured directly for the entire samples range from 13 ± 1 to 895 ± 9 Bq m^−3^ with an average value of 116 ± 7 Bq m^−3^. The lowest ^222^Rn activity concentration came from the brick sample (J3) from the Parung area. In contrast, the highest value came from the 10 cm thick AAC sample (I4) obtained from the Cimanggu area. The ^222^Rn activity concentration values obtained from the direct measurements were used to calculate the ^222^Rn surface exhalation rates by applying Equation 1. The distribution of the ^222^Rn surface exhalation rate for all the building materials studied is presented in Figure 4. The principal data obtained for each building material type, sand, cement, brick, 10 cm AAC, and 7 cm AAC was presented in Table 2. The ^222^Rn surface exhalation rate ranges from 4 × 10^−2^ to 1.6 × 10^−0^ mBq m^−2^ s^−1^ with an average of 4 × 10^−1^ mBq m^−2^ s^−1^.

**Table 2:** Principal data of ^222^Rn activity concentration and surface exhalation rate.

Building material	Statistical parameter	^222^Rn activity concentration (Bq m^−3^)	^222^Rn surface exhalation rate (mBq m^−2^ s^−1^)
Cement	Minimum	52	2 × 10^−1^
	Maximum	209	7 × 10^−1^
	Average	114	4 × 10^−1^
Brick	Minimum	13	4 × 10^−2^
	Maximum	193	8 × 10^−1^
	Average	44	3 × 10^−1^
Sand	Minimum	47	1 × 10^−1^
	Maximum	441	6 × 10^−1^
	Average	109	2 × 10^−1^
10 cm AAC	Minimum	20	7 × 10^−2^
	Maximum	895	2 × 10^0^
	Average	198	6 × 10^−1^
7 cm AAC	Minimum	27	8 × 10^−2^
	Maximum	674	1 × 10^0^
	Average	159	6 × 10^−1^

### 3.2 Activity concentration of ^226^Ra, ^232^Th, and ^40^K in building materials

The results obtained from gamma spectrometry analysis of the building materials to determine ^226^Ra, ^232^Th, and ^40^K activity concentrations were presented in Table 3. The ^226^Ra activity concentration values ranged from 4 ± 0.2 to 107 ± 4 Bq kg^−1^; ^232^Th activity concentration ranged from 1 ± 0.1 to 85 ± 3 Bq kg^−1^; and ^40^K activity concentration ranged from 20 ± 1 to 940 ± 26 Bq kg^−1^. The lowest ^226^Ra activity concentration came from the 10-cm thick AAC sample (A4) obtained from the Pondok Cabe area. In comparison, the highest value came from the 7-cm thick AAC sample (I5) obtained from the Cimanggu area, and the lowest ^232^Th activity concentration came from the 7-cm thick AAC sample (A5) obtained from the Pondok Cabe area. In comparison, the highest ^232^Th activity concentration came from a sand sample (N1) obtained from the Bambu Apus area, and the lowest ^40^K activity concentration came from a 7-cm thick AAC sample (A5) obtained from the Pondok Cabe area. In contrast, the highest ^40^K activity concentration came from a sand sample (H1) obtained from the Lebak Bulus area.

**Table 3:** ^226^Ra, ^232^Th, and ^40^K activity concentrations were measured in the building materials.

Building material	Statistical parameter	Activity concentration (Bq kg^−1^)		
		^226^Ra	^232^Th	^40^K
Cement	Range	9–86	2–22	22–212
	Average	49	14	127
	Median	51	16	127
	Sample number	16	16	16
Brick	Range	17–47	27–61	127–394
	Average	35	43	213
	Median	34	44	192
	Sample number	13	13	13
Sand	Range	4–91	6–85	59–940
	Average	31	37	473
	Median	29	31	489
	Sample number	19	19	
10-cm AAC	Range	4–73	3–56	42–539
	Average	43	22	289
	Median	41	20	258
	Sample number	17	17	17
7-cm AAC	Range	5–107	1–34	20–512
	Average	50	50	286
	Median	46	46	319
	Sample number	16	16	16

### 3.3 Indoor ^222^Rn activity concentration originated from the building materials, activity index, and annual effective dose received by an adult individual from different exposure pathways

Using the typical room size in the study area and the average ^226^Ra content of the building materials, the contribution of the building materials to the indoor ^222^Rn activity concentration was calculated to be 28 Bq m^−3^. The activity index was calculated and ranged from 0.2 to 0.5, with an average of 0.3. The highest value of the activity index was obtained from 7-cm AAC, while the lowest value came from cement. Furthermore, the annual effective doses from different exposure pathways for various building materials calculated using the RESRAD-BUILD computer code are presented in Table 4. In a realistic situation, a typical house constructed with a mixture of building materials generated annual effective doses of 0.01 mSv by external directly from the source, 1.6 × 10^−6^ mSv by ingestion from deposition, and 0.1 mSv from ^222^Rn. The total annual effective derived from these exposures in the typical house is 0.11 mSv.

**Table 4:** The annual effective dose received by an individual living in the house from the building materials studied.

Building material	Annual effective dose (mSv)			
	External	Ingestion	^222^Rn	Total
Cement	5.7 × 10^−3^	3.4 × 10^−6^	8 × 10^−2^	8.2 × 10^−2^
Brick	5.0 × 10^−3^	2.4× 10^−7^	6.0 × 10^−2^	6.4 × 10^−2^
Sand	4.4 × 10^−3^	5.9 × 10^−6^	6.6 × 10^−2^	7.0 × 10^−2^
10 cm AAC	6.1 × 10^−3^	3.2 × 10^−7^	7.8 × 10^−2^	8.4 × 10^−2^
7 cm AAC	6.1 × 10^−3^	3.2 × 10^−7^	7.8 × 10^−2^	8.4 × 10^−2^
Typical house*	9.1 × 10^−3^	1.6 × 10^−6^	1.0 × 10^−1^	1.1 × 10^−1^

*A house of 50% bricks, 30% cement, and 20% sand.

## 4 Discussion

### 4.1 ^222^Rn surface exhalation rates in building materials

**Figure 5:**
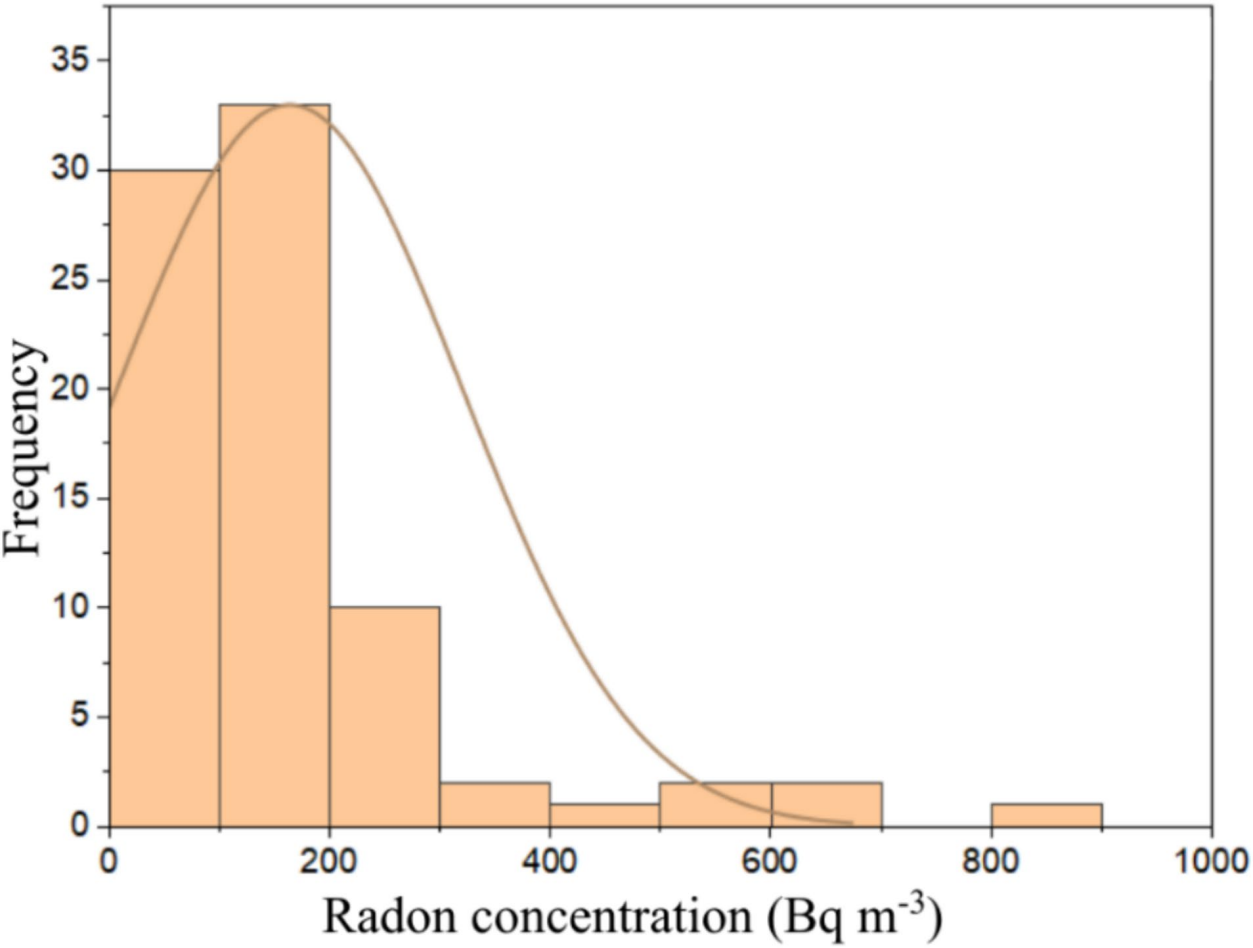
Frequency distribution of ^222^Rn activity concentration in building materials.

The average ^222^Rn surface exhalation rate values of the building materials studied were lower than the global average value estimated at 1.6 × 10^1^ mBq m^−2^ s^−1^ (45). Surface exhalation rate measurements are essential to determine potential high-risk areas due to ^222^Rn inhalation (35). Among the all-studied building materials, the lowest ^222^Rn surface exhalation rate value comes from a brick sample (E3) from Serpong. In contrast, the highest value comes from a 10-cm thick AAC sample (I4) from Cimanggu. The ^222^Rn surface exhalation rate value varies from one sample to another. Factors that may influence the ^222^Rn exhalation rate are the level of ^226^Ra content in the material for ^222^Rn surface exhalation rate, grain size, porosity, humidity, ^222^Rn diffusion in the material’s pores, and changes in pressure and material texture (47). The ^222^Rn surface exhalation rate of building material with other countries was presented in Table 5. For comparison purposes, the ^222^Rn exhalation rate of bricks for selected countries was expressed in Bq m-2 h-1. It is noted that the results obtained in this remain lower than those of Turkey, Saudi Arabia, and India (48–50), but in the range of the results found in Ecuador (30). The ^222^Rn surface exhalation rate level does not pose a significant risk to the public regarding the ^222^Rn exhalation rate. Furthermore, the frequency distribution of ^222^Rn activity concentration was shown in Figure 5, and was tested for its normality by using the Kolmogorov–Smirnov test. The Kolmogorov–Smirnov test can be used for more than 50 samples. The conditions that must be met when carrying out the Kolmogorov–Smirnov test are if the significance value is more than 0.05, then the data used in the research has a normal distribution. However, on the contrary, if the significance value is less than 0.05, then the data used does not have a normal distribution (51).

**Table 5:** Comparison of ^222^Rn surface exhalation rate of building material with other countries.

Country	^222^Rn surface exhalation rate (Bq m^−2^ h^−1^)	
India	224	(49)
Turkey	129	(51)
Ecuador	<0.5	(30)
Saudi Arabia	37	(50)
Indonesia	1.3	This study

### 4.2 Activity concentration of natural radionuclides in the building materials

The average activity concentration values of ^232^Th (21 Bq kg^−1^) and ^40^K (217 Bq kg^−1^) in all building materials studied did not exceed the global average values of 45 Bq kg^−1^ and 412 Bq kg^−1^. In comparison, the average values of the activity concentration of ^226^Ra (34 Bq kg^−1^) are close to the global average value of 32 Bq kg^−1^ based on UNSCEAR reports (4). A comparison between the results of ^226^Ra, ^232^Th, and ^40^K activity concentrations in this study with the results of similar studies reported in various countries was shown in Table 6. In this study, the highest ^226^Ra activity concentration value was obtained at 42 Bq kg^−1^, lower than Italy and Malaysia; the highest ^232^Th activity was 42 Bq kg^−1^, lower than India, China, and Malaysia, while the highest ^40^K activity was 390 Bq kg^−1^ lower than Malaysia, China, Iran, Italy. The concentration values of natural radionuclides in building materials vary from one country to another. Variations in the activity concentration values of natural radionuclide activity may be caused by differences in mineral content in the soil and the geographical origin of the raw materials (17). The high activity concentration of ^226^Ra indicates the high uranium activity concentration in the soil and rocks in the raw material source area. The high activity concentration of ^226^Ra indicates the high uranium concentration in the soil and rocks in the raw material source area. A high activity concentration of ^226^Ra will have the potential for high ^222^Rn gas released by the material. High ^232^Th activity concentration activity will have the potential for high levels of thoron gas released by the material (24). On the results of research that has been carried out, the activity concentrations of ^226^Ra, ^232^Th, and ^40^K in sand samples (D1, G1, H1, and L1) and 10 cm thick AAC (N3) have values higher than the global average according to UNSCEAR (4). Sand samples with natural radionuclide concentrations exceeding the global average were obtained from different regions but came from the same mining area, namely Rangkasbitung. According to Lebak Regency Regional Regulation Number 2 of 2014, there are 25 sand mineral mining areas in Lebak Regency, one of which is the Rangkasbitung area (51). AAC samples with natural radionuclide concentrations higher than the global average come from the Tangerang, Gunung Sindur, and Serang areas. The high activity concentration values of ^226^Ra, ^232^Th, and ^40^K in these materials do not come from contamination but from natural radionuclides in the source materials.

**Table 6:** Comparison of building materials with other countries.

Country	*N*	Mean activity concentration (Bq kg ^1^)			Reference
		^226^Ra	^232^Th	^40^K	
Sand					
Malaysia	10	43	45	451	(23)
Egypt	3	17	13	119	(53)
Qatar	4	13	3	225	(54)
Meghalaya, India	11	3.7	40	263	(51)
India	7	11	130	297	(55)
Iran	3	24	22	362	(25)
Cameroon	16	32	54	443	(16)
Indonesia	19	24	31	390	This study
Cement					
Malaysia	10	29	31	205	(23)
Arab Saudi	4	22	10	102	(56)
Qatar	6	23	10	120	(54)
Meghalaya, India	7	47	57	312	(50)
India	3	37	34	188	(55)
Cameroon	7	26	18	173	(16)
Indonesia	16	42	11	107	This study
Brick					
Malaysia	5	40	58	556	(23)
China	4	14	39	678	(57)
Meghalaya, India	5	53	63	405	(50)
Iran	77	37	12	851	(25)
Italy	7	58	51	473	(58)
Poland	39	50	50	963	(59)
Indonesia	13	34	42	202	This study
AAC					
China	4	16	51	605	(57)
Indonesia	17	38	18	239	This study

**Figure 6:**
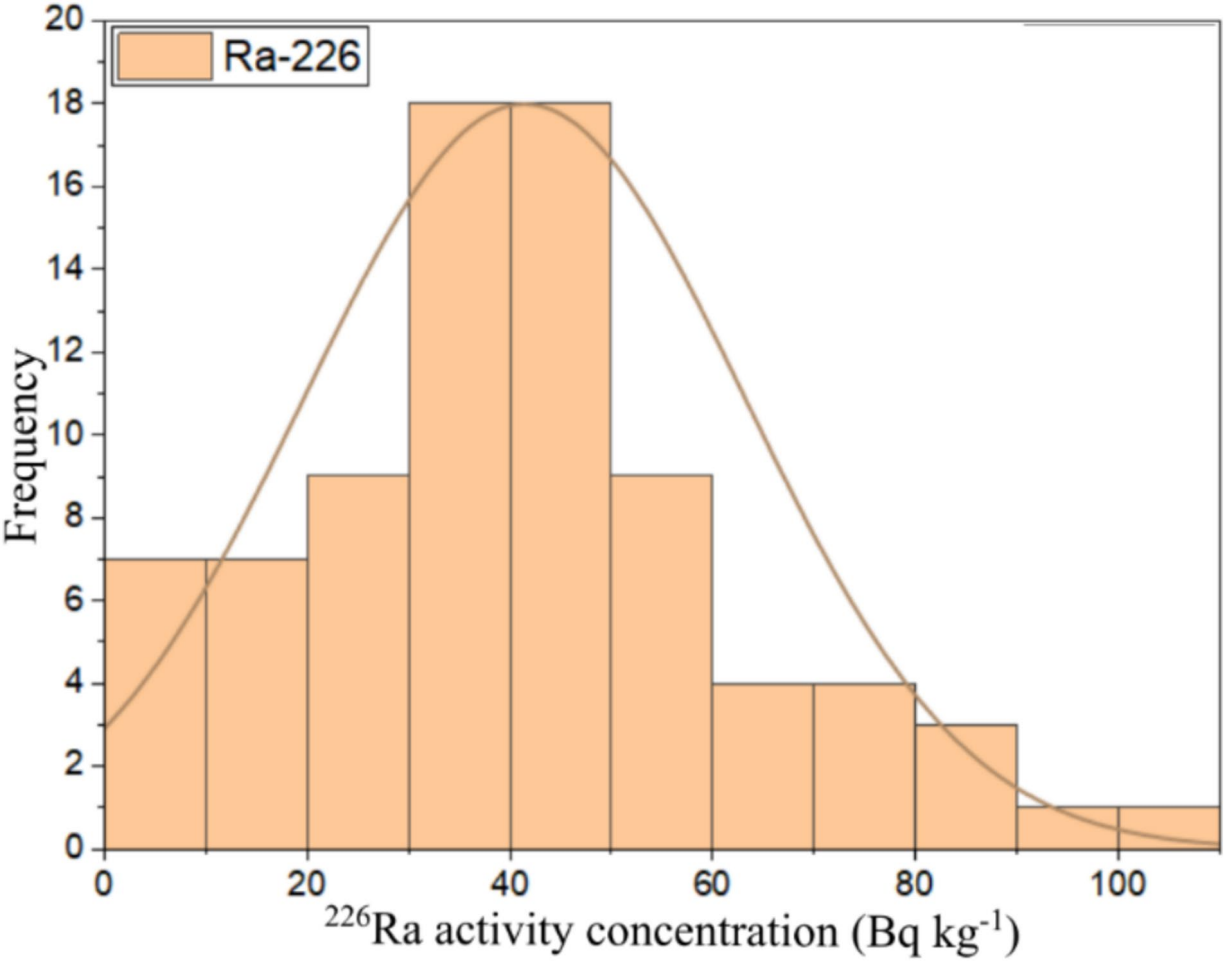
Frequency distribution of ^226^Ra activity concentration in building materials.

**Figure 7:**
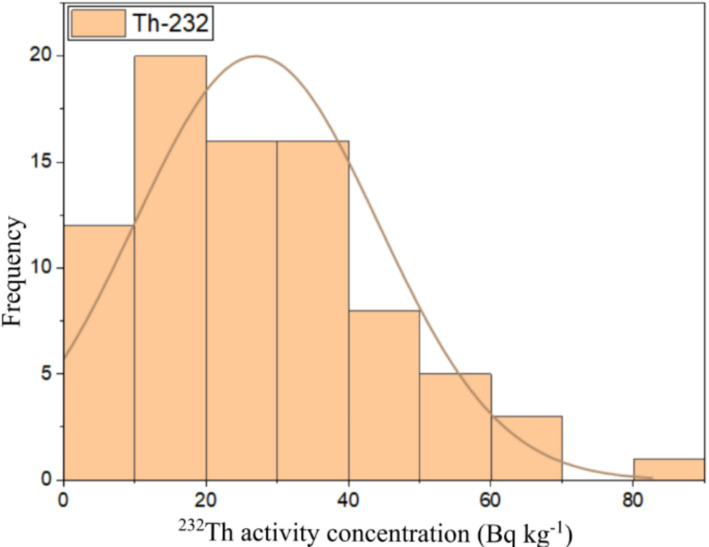
Frequency distribution of ^232^Th activity concentration in building materials.

**Figure 8:**
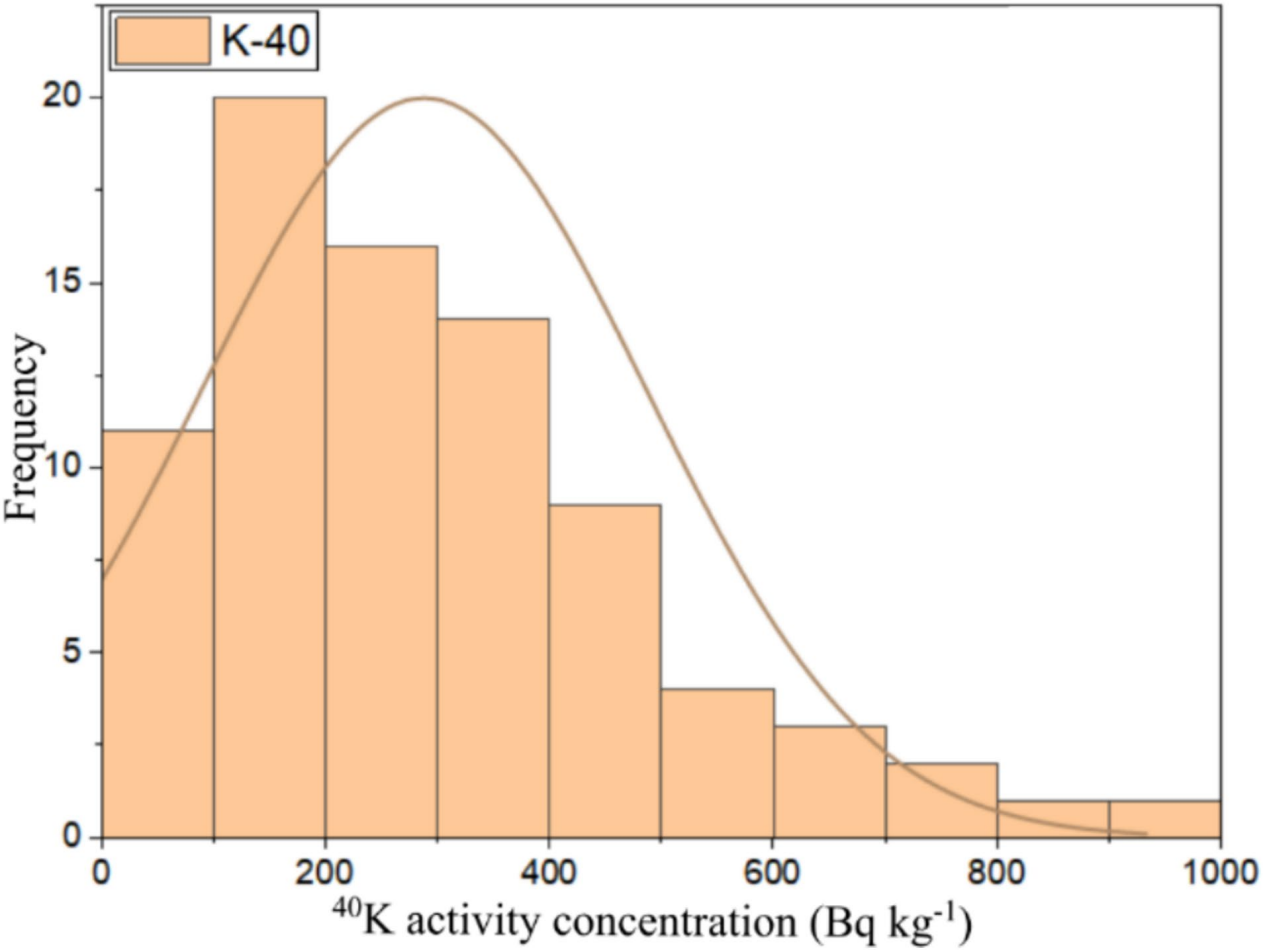
Frequency distribution of ^40^K activity concentration in building materials.

This study used numerical and graphical methods to investigate the normality distribution of ^226^Ra, ^232^Th, and ^40^K activity concentrations in building material samples. All statistical analyses were performed using ORIGIN PRO 2023 software (student version). The frequency distribution of radionuclides in building materials was assessed using the Kolmogorov–Smirnov normality test with significance values of 0.46, 0.67, and 0.06 for ^226^Ra, ^232^Th, and ^40^K, respectively. As all the significance values were more than 0.05, all the radionuclide distributions had a normal distribution. The graphical representation of the data distributions of ^226^Ra, ^232^Th, and ^40^K were presented in the form of histograms shown in Figures 6–8, respectively. It can be seen that ^226^Ra, ^232^Th, and ^40^K are distributed normally or lognormally.

### 4.3 Indoor ^222^Rn from building material, activity index, and effective dose from different pathways

The indoor ^222^Rn concentration derived from building materials calculated in this study is lower than the global average value of 40 Bq m^−3^, considerably less than the WHO reference value of 100 Bq m^−3^, and the Indonesian national reference level of 300 Bq m^−3^ (52). However, it exceeds the values obtained by Ndjana et al., where the authors reported values of 10 and 7 Bq m^−3^ from building materials (16). Therefore, a large indoor ^222^Rn survey should be undertaken in future studies. As presented in the results section, the effective dose from ^222^Rn represents 92% of the total effective inhalation dose. The activity index introduced by the IAEA to assess the radiological risk of natural radionuclides contained in building materials, as calculated in this study, was low. The activity index average value of 0.3 was below the reference value of 1 and does not require any restriction from their use (43). Also, the total annual effective doses from different exposure pathways calculated presented values ranging from 6.4 × 10^−2^ to 1.1 × 10^−1^ mSv depending on the building materials type. For the typical house built using a mixture of building materials, the total annual effective dose of 0.11 mSv was less than the global average value of 2.4 mSv, which resulted from all natural sources combined. This total annual effective dose is in the range of the results found in Ecuador by Tene et al. while studying the radiological exposure from building materials, and the range was from 0.019 up to 0.112 mSv (30).

## 5 Conclusion

In this study, a safety assessment of radiological hazards in building materials was conducted to ensure a sustainable environment. 81 building material samples were collected from 17 regional areas in Jakarta and its surrounding areas. The ^222^Rn surface exhalation rate from the collected building materials ranges from 4 × 10^−2^ to 1.6 × 10^0^ mBq m^−2^ s^−1^ with an average of 4 × 10^−1^ mBq m^−2^ s^−1^. The average ^222^Rn surface exhalation rate of the building materials studied was much lower than the global average value of 16 mBq m^−2^ s^−1^ (45). The average activity concentration values of ^232^Th (21 Bq kg^−1^) and ^40^K (217 Bq kg^−1^) in all building materials studied are lower than the global average values of 45 Bq kg^−1^ and 412 Bq kg^−1^. In comparison, the average value of the activity concentration of ^226^Ra (34 Bq kg^−1^) is close to the global average value of 32 Bq kg^−1^ (4). Additionally, indoor ^222^Rn derived from building materials was calculated along with the activity index (I), and the computer code RESRAD-BUILD was used to estimate the total annual effective dose received from different exposure pathways for a typical house built using a mixture of building materials. It was found that the average activity index was below unity, and the total annual effective dose received was 0.11 mSv, in which ^222^Rn alone contributes to 92% of this value, which indicates the need for a large indoor ^222^Rn survey in the study area. In line with the SDGs defined by the United Nations, the building materials studied presented radionuclide concentrations and the activity index below the reference values of remediation action to be taken. From a radiological exposure perspective, these building materials are considered safe for building homes, offices, industries, and infrastructure.

## Author’s note

*What are the main findings?* (1) The ^222^Rn exhalation rate ranges from 4 × 10−2 to 1.6 × 100 mBq m−2 s−1 with an average of 4 × 10−1 mBq m−2 s−1. The average activity concentration values of 226Ra, 232Th, and 40K were 34, 21, and 217 Bq kg−1, respectively, across all the building materials studied. (2) Furthermore, indoor ^222^Rn was derived from building materials, and the activity index was calculated. The computer code RESRAD-BUILD was used to calculate the annual effective dose received from different exposure pathways. The total annual effective dose received in a typical house constructed with a mixture of building materials was 0.11 mSv, and ^222^Rn alone contributed 92% of the total. *What is the implication of the main finding?* (1) From the measurement results, it was found that the building materials in Jakarta and its surrounding areas do not possess significant concerns regarding radioactivity. The building materials we studied showed radionuclide concentrations below the recommended reference values for remediation action, aligning perfectly with the SDGs. From a radiological exposure perspective, these building materials are considered safe for building homes, offices, industries, and infrastructure. (2) The high contribution of ^222^Rn to the total annual effective dose indicates the need for a large indoor ^222^Rn survey in the study area. Furthermore, this study can aid the regulatory body’s willingness to establish standards in the construction sector.

## Data Availability

The original contributions presented in the study are included in the article/Supplementary material, further inquiries can be directed to the corresponding author.
