# Supplementary material for: Radon exhalation rate and natural radioactivity in the building materials used in metropolitan Jakarta and its surrounding areas, Indonesia
Source: Front Public Health. 2025 Feb 18;13:1539957. doi: 10.3389/fpubh.2025.1539957 (PMC11877393; doi:10.3389/fpubh.2025.1539957)
Supplement: Supplementary file 1 [file Data_Sheet_1.pdf]

Title : Default Case for RESRAD-BUILD

Input File : C:\RESRAD_Family\BUILD\4.0\UserFiles\OBM TEST 1.bld

RESRAD-BUILD Table of Contents

RESRAD-BUILD Input Parameters............ 2

Building Information..................... 3

Source Information....................... 4

Temporal Dose Summary.................... 9

For time = 0.00E+00 yr

  Receptor-Source Dose Summary.......... 10

  Dose by Pathway Detail................ 11

  Dose by Nuclide Detail................ 12

For time = 1.00E+00 yr

  Receptor-Source Dose Summary.......... 14

  Dose by Pathway Detail................ 15

  Dose by Nuclide Detail................ 16

Title : Default Case for RESRAD-BUILD

Input File : C:\RESRAD_Family\BUILD\4.0\UserFiles\OBM TEST 1.bld

RESRAD-BUILD Input Parameters

Number of Sources : 5

Number of Receptors: 1

Total Time : 3.650000E+02 days

Fraction Inside : 8.000000E-01

Transformations data from: ICRP38

Cut off half life : 30.0000000 days

Library information in the Dose Coefficients and Slope Factors report

Receptor Information

Receptor	Room	x [m]	y [m]	z [m]	FracTime	Inhalation [m3/day]	Ingestion(Dust) [m2/hr]
1	1	2.000	2.000	1.500	1.000	2.00E+01	1.00E-04

Receptor-Source Shielding Relationship

Receptor	Source	Density [g/cm3]	Thickness [cm]	Material
1	1	2.40E+00	1.50E+01	Concrete
1	2	2.40E+00	1.50E+01	Concrete
1	3	2.40E+00	1.50E+01	Concrete
1	4	2.40E+00	1.50E+01	Concrete
1	5	2.40E+00	1.50E+01	Concrete

Building Information

Building Air Exchange Rate: 8.00E-01 1/hr

```
   Height[m]         Air Exchanges [m3/hr]
   Area [m2]
                    ******************************
                    *                            *
                    *                            *
                    *                          <=Q01: 0.00E+00
   H1:   2.500      *           Room  1          *      Q10 :  0.00E+00
                    *   LAMBDA: 0.00E+00         *
  Area  36.000      *                            *
                    *                            *
                    ******************************
```

Deposition velocity: 1.00E-02 [m/s]   Resuspension Rate: 0.00E+00 [1/s]

Deposition velocity: 5.00E-07 [m/s]   Resuspension Rate:

Source Information

Source: 1

Location:: Room : 1 x: 2.00 y: 2.00 z: 0.00[m]

Geometry:: Type: Volume Length[m]:4.00E+00 Width[m]:4.00E+00 Direction: -z

Pathway ::

Direct Ingestion Rate: 0.000E+00 [gm/hr]

Fraction released to air: 1.000E-01

Containment :: Number of Regions: 4 Contaminated Region: 1

Region	1	2	3	4
Thickness [cm]	1.50E+01	1.50E+01	1.50E+01	1.50E+01
Density [g/cm3]	2.40E+00	2.40E+00	2.40E+00	2.40E+00
Material	Concrete			
Erosion Rate [cm/day]	2.40E-08	2.40E-08	2.40E-08	2.40E-08
Porosity	1.00E-01	1.00E-01	1.00E-01	1.00E-01
Eff. Diffusion [m2/s]	2.00E-05	2.00E-05	2.00E-05	2.00E-05
Emanation Fractions(1)	2.00E-01	2.00E-01	2.00E-01	2.00E-01
(2)	3.80E-01	2.00E-01	2.00E-01	2.00E-01

Contamination::

Nuclide	Concentration [Bq /g]
K-40	1.270E-01
Ra-226	4.900E-02
Th-232	1.400E-02

```
Source:  2
        Location::  Room :  1  x:   0.00 y:   2.00  z:   2.00[m]
        Geometry::  Type: Volume      Length[m]:4.00E+00  Width[m]:3.00E+00  Direction: -x
        Pathway ::
             Direct Ingestion Rate:    0.000E+00 [gm/hr]
             Fraction released to air: 1.000E-01



        Containment ::   Number of Regions:  4 Contaminated Region:  1
    Region                :    1        2        3        4
    Thickness [cm]        :1.50E+01 1.50E+01 1.50E+01 1.50E+01
    Density [g/cm3]       :2.40E+00 2.40E+00 2.40E+00 2.40E+00
    Material              :Concrete
    Erosion Rate [cm/day] :2.40E-08 2.40E-08 2.40E-08 2.40E-08
    Porosity              :1.00E-01 1.00E-01 1.00E-01 1.00E-01
    Eff. Diffusion [m2/s] :2.00E-05 2.00E-05 2.00E-05 2.00E-05
    Emanation Fractions(1):2.00E-01 2.00E-01 2.00E-01 2.00E-01
                       (2):3.80E-01 2.00E-01 2.00E-01 2.00E-01

   Contamination::
   Nuclide Concentration
   _______ _____________
                [Bq /g]
    K-40       1.270E-01
    Ra-226     4.900E-02
    Th-232     1.400E-02
```

Title : Default Case for RESRAD-BUILD

Input File : C:\RESRAD_Family\BUILD\4.0\UserFiles\OBM TEST 1.bld

Source: 3

Location:: Room : 1 x: 2.00 y: 0.00 z: 2.00[m]

Geometry:: Type: Volume   Length[m]:4.00E+00 Width[m]:3.00E+00 Direction: -y

Pathway ::

Direct Ingestion Rate: 0.000E+00 [gm/hr]

Fraction released to air: 1.000E-01

Containment ::   Number of Regions: 4 Contaminated Region: 1

Region	1	2	3	4
Thickness [cm]	1.50E+01	1.50E+01	1.50E+01	1.50E+01
Density [g/cm3]	2.40E+00	2.40E+00	2.40E+00	2.40E+00
Material	Concrete			
Erosion Rate [cm/day]	2.40E-08	2.40E-08	2.40E-08	2.40E-08
Porosity	1.00E-01	1.00E-01	1.00E-01	1.00E-01
Eff. Diffusion [m2/s]	2.00E-05	2.00E-05	2.00E-05	2.00E-05
Emanation Fractions(1)	2.00E-01	2.00E-01	2.00E-01	2.00E-01
(2)	3.80E-01	2.00E-01	2.00E-01	2.00E-01

Contamination::

Nuclide	Concentration
	[Bq /g]
K-40	1.270E-01
Ra-226	4.900E-02
Th-232	1.400E-02

Title : Default Case for RESRAD-BUILD
Input File : C:\RESRAD_Family\BUILD\4.0\UserFiles\OBM TEST 1.bld

Source: 4

Location:: Room : 1 x: 2.00 y: 4.00 z: 2.00[m]

Geometry:: Type: Volume   Length[m]:4.00E+00 Width[m]:3.00E+00 Direction: -y

Pathway ::

Direct Ingestion Rate: 0.000E+00 [gm/hr]

Fraction released to air: 1.000E-01

Containment ::   Number of Regions: 4 Contaminated Region: 1

Region	1	2	3	4
Thickness [cm]	1.50E+01	1.50E+01	1.50E+01	1.50E+01
Density [g/cm3]	2.40E+00	2.40E+00	2.40E+00	2.40E+00
Material	Concrete			
Erosion Rate [cm/day]	2.40E-08	2.40E-08	2.40E-08	2.40E-08
Porosity	1.00E-01	1.00E-01	1.00E-01	1.00E-01
Eff. Diffusion [m2/s]	2.00E-05	2.00E-05	2.00E-05	2.00E-05
Emanation Fractions(1)	2.00E-01	2.00E-01	2.00E-01	2.00E-01
(2)	3.80E-01	2.00E-01	2.00E-01	2.00E-01

Contamination::

Nuclide	Concentration
	[Bq /g]
K-40	1.270E-01
Ra-226	4.900E-02
Th-232	1.400E-02

Title : Default Case for RESRAD-BUILD

Input File : C:\RESRAD_Family\BUILD\4.0\UserFiles\OBM TEST 1.bld

```
Source:  5
        Location::  Room :  1  x:   4.00 y:   2.00  z:   2.00[m]
        Geometry::  Type: Volume      Length[m]:4.00E+00  Width[m]:3.00E+00  Direction: -x
        Pathway ::
             Direct Ingestion Rate:    0.000E+00 [gm/hr]
             Fraction released to air: 1.000E-01



        Containment ::   Number of Regions:  4 Contaminated Region:  1
    Region                :    1        2        3        4
    Thickness [cm]        :1.50E+01 1.50E+01 1.50E+01 1.50E+01
    Density [g/cm3]       :2.40E+00 2.40E+00 2.40E+00 2.40E+00
    Material              :Concrete
    Erosion Rate [cm/day] :2.40E-08 2.40E-08 2.40E-08 2.40E-08
    Porosity              :1.00E-01 1.00E-01 1.00E-01 1.00E-01
    Eff. Diffusion [m2/s] :2.00E-05 2.00E-05 2.00E-05 2.00E-05
    Emanation Fractions(1):2.00E-01 2.00E-01 2.00E-01 2.00E-01
                       (2):3.80E-01 2.00E-01 2.00E-01 2.00E-01

   Contamination::
   Nuclide Concentration
   _______ _____________
                [Bq /g]
    K-40       1.270E-01
    Ra-226     4.900E-02
    Th-232     1.400E-02
```

Title : Default Case for RESRAD-BUILD

Input File : C:\RESRAD_Family\BUILD\4.0\UserFiles\OBM TEST 1.bld

Temporal Summary

RESRAD-BUILD Temporal Dose Tables

Dose to Receptor over the Exposure Duration

(mSv)

Evaluation Time years	Receptor 1	Total
0.000000000	1.01E-01	1.01E-01
1.000000000	1.13E-01	1.13E-01

Title : Default Case for RESRAD-BUILD

Input File : C:\RESRAD_Family\BUILD\4.0\UserFiles\OBM TEST 1.bld

Evaluation Time: 0.00000000E+00 years

RESRAD-BUILD Dose Tables

Source Contributions to Receptor Doses

[mSv]

	Source 1	Source 2	Source 3	Source 4	Source 5	Total
Receptor 1	2.29E-02	1.72E-02	1.72E-02	2.17E-02	2.17E-02	1.01E-01
Total	2.29E-02	1.72E-02	1.72E-02	2.17E-02	2.17E-02	1.01E-01

Title : Default Case for RESRAD-BUILD

Input File : C:\RESRAD_Family\BUILD\4.0\UserFiles\OBM TEST 1.bld

Evaluation Time: 0.00000000E+00 years

Pathway Detail of Doses

[mSv]

Source: 1

Receptor	External directly from Source	Inhalation	External from Deposition on floor	External from Suspension in air	Ingestion of Deposition	Radon	Ingestion of Source	Total
1	1.99E-06	2.26E-06	2.19E-09	2.48E-11	4.71E-08	2.29E-02	0.00E+00	2.29E-02
Total	1.99E-06	2.26E-06	2.19E-09	2.48E-11	4.71E-08	2.29E-02	0.00E+00	2.29E-02

Source: 2

Receptor	External directly from Source	Inhalation	External from Deposition on floor	External from Suspension in air	Ingestion of Deposition	Radon	Ingestion of Source	Total
1	1.80E-06	1.69E-06	1.65E-09	1.86E-11	3.53E-08	1.72E-02	0.00E+00	1.72E-02
Total	1.80E-06	1.69E-06	1.65E-09	1.86E-11	3.53E-08	1.72E-02	0.00E+00	1.72E-02

Source: 3

Receptor	External directly from Source	Inhalation	External from Deposition on floor	External from Suspension in air	Ingestion of Deposition	Radon	Ingestion of Source	Total
1	1.80E-06	1.69E-06	1.65E-09	1.86E-11	3.53E-08	1.72E-02	0.00E+00	1.72E-02
Total	1.80E-06	1.69E-06	1.65E-09	1.86E-11	3.53E-08	1.72E-02	0.00E+00	1.72E-02

Source: 4

Receptor	External directly from Source	Inhalation	External from Deposition on floor	External from Suspension in air	Ingestion of Deposition	Radon	Ingestion of Source	Total
1	4.47E-03	1.69E-06	1.65E-09	1.86E-11	3.53E-08	1.72E-02	0.00E+00	2.17E-02
Total	4.47E-03	1.69E-06	1.65E-09	1.86E-11	3.53E-08	1.72E-02	0.00E+00	2.17E-02

Source: 5

Receptor	External directly from Source	Inhalation	External from Deposition on floor	External from Suspension in air	Ingestion of Deposition	Radon	Ingestion of Source	Total
1	4.47E-03	1.69E-06	1.65E-09	1.86E-11	3.53E-08	1.72E-02	0.00E+00	2.17E-02
Total	4.47E-03	1.69E-06	1.65E-09	1.86E-11	3.53E-08	1.72E-02	0.00E+00	2.17E-02

Title : Default Case for RESRAD-BUILD

Input File : C:\RESRAD_Family\BUILD\4.0\UserFiles\OBM TEST 1.bld

Evaluation Time: 0.00000000E+00 years

Nuclide Detail of Doses

[mSv]

Source: 1

Nuclide	Receptor 1	Total
K-40	4.98E-07	4.98E-07
Ra-226	2.23E-02	2.23E-02
Pb-210	7.53E-09	7.53E-09
Po-210	3.19E-09	3.19E-09
Th-232	1.72E-06	1.72E-06
Ra-228	2.48E-08	2.48E-08
Th-228	6.14E-04	6.14E-04

Source: 2

Nuclide	Receptor 1	Total
K-40	4.50E-07	4.50E-07
Ra-226	1.67E-02	1.67E-02
Pb-210	5.65E-09	5.65E-09
Po-210	2.40E-09	2.40E-09
Th-232	1.29E-06	1.29E-06
Ra-228	1.95E-08	1.95E-08
Th-228	4.60E-04	4.60E-04

Source: 3

Nuclide	Receptor 1	Total
K-40	4.50E-07	4.50E-07
Ra-226	1.67E-02	1.67E-02
Pb-210	5.65E-09	5.65E-09
Po-210	2.40E-09	2.40E-09
Th-232	1.29E-06	1.29E-06
Ra-228	1.95E-08	1.95E-08
Th-228	4.60E-04	4.60E-04

Title : Default Case for RESRAD-BUILD

Input File : C:\RESRAD_Family\BUILD\4.0\UserFiles\OBM TEST 1.bld

Evaluation Time: 0.00000000E+00 years

Source: 4

Nuclide	Receptor 1	Total
K-40	9.59E-04	9.59E-04
Ra-226	2.02E-02	2.02E-02
Pb-210	8.22E-09	8.22E-09
Po-210	2.48E-09	2.48E-09
Th-232	1.29E-06	1.29E-06
Ra-228	2.74E-05	2.74E-05
Th-228	4.67E-04	4.67E-04

Source: 5

Nuclide	Receptor 1	Total
K-40	9.59E-04	9.59E-04
Ra-226	2.02E-02	2.02E-02
Pb-210	8.22E-09	8.22E-09
Po-210	2.48E-09	2.48E-09
Th-232	1.29E-06	1.29E-06
Ra-228	2.74E-05	2.74E-05
Th-228	4.67E-04	4.67E-04

Title : Default Case for RESRAD-BUILD

Input File : C:\RESRAD_Family\BUILD\4.0\UserFiles\OBM TEST 1.bld

Evaluation Time: 1.00000000 years

RESRAD-BUILD Dose Tables

Source Contributions to Receptor Doses

[mSv]

	Source 1	Source 2	Source 3	Source 4	Source 5	Total
Receptor 1	2.61E-02	1.96E-02	1.96E-02	2.41E-02	2.41E-02	1.13E-01
Total	2.61E-02	1.96E-02	1.96E-02	2.41E-02	2.41E-02	1.13E-01

Title : Default Case for RESRAD-BUILD

Input File : C:\RESRAD_Family\BUILD\4.0\UserFiles\OBM TEST 1.bld

Evaluation Time: 1.00000000 years

Pathway Detail of Doses

[mSv]

Source: 1

Receptor	External directly from Source	Inhalation	External from Deposition on floor	External from Suspension in air	Ingestion of Deposition	Radon	Ingestion of Source	Total
1	2.05E-06	4.68E-06	4.90E-09	5.10E-11	1.20E-07	2.61E-02	0.00E+00	2.61E-02
Total	2.05E-06	4.68E-06	4.90E-09	5.10E-11	1.20E-07	2.61E-02	0.00E+00	2.61E-02

Source: 2

Receptor	External directly from Source	Inhalation	External from Deposition on floor	External from Suspension in air	Ingestion of Deposition	Radon	Ingestion of Source	Total
1	1.85E-06	3.51E-06	3.68E-09	3.82E-11	9.01E-08	1.96E-02	0.00E+00	1.96E-02
Total	1.85E-06	3.51E-06	3.68E-09	3.82E-11	9.01E-08	1.96E-02	0.00E+00	1.96E-02

Source: 3

Receptor	External directly from Source	Inhalation	External from Deposition on floor	External from Suspension in air	Ingestion of Deposition	Radon	Ingestion of Source	Total
1	1.85E-06	3.51E-06	3.68E-09	3.82E-11	9.01E-08	1.96E-02	0.00E+00	1.96E-02
Total	1.85E-06	3.51E-06	3.68E-09	3.82E-11	9.01E-08	1.96E-02	0.00E+00	1.96E-02

Source: 4

Receptor	External directly from Source	Inhalation	External from Deposition on floor	External from Suspension in air	Ingestion of Deposition	Radon	Ingestion of Source	Total
1	4.56E-03	3.51E-06	3.68E-09	3.82E-11	9.01E-08	1.96E-02	0.00E+00	2.41E-02
Total	4.56E-03	3.51E-06	3.68E-09	3.82E-11	9.01E-08	1.96E-02	0.00E+00	2.41E-02

Source: 5

Receptor	External directly from Source	Inhalation	External from Deposition on floor	External from Suspension in air	Ingestion of Deposition	Radon	Ingestion of Source	Total
1	4.56E-03	3.51E-06	3.68E-09	3.82E-11	9.01E-08	1.96E-02	0.00E+00	2.41E-02
Total	4.56E-03	3.51E-06	3.68E-09	3.82E-11	9.01E-08	1.96E-02	0.00E+00	2.41E-02

Title : Default Case for RESRAD-BUILD

Input File : C:\RESRAD_Family\BUILD\4.0\UserFiles\OBM TEST 1.bld

Evaluation Time: 1.00000000 years

Nuclide Detail of Doses

[mSv]

Source: 1

Nuclide	Receptor 1	Total
K-40	5.01E-07	5.01E-07
Ra-226	2.23E-02	2.23E-02
Pb-210	3.72E-08	3.72E-08
Po-210	2.43E-08	2.43E-08
Th-232	3.47E-06	3.47E-06
Ra-228	1.08E-07	1.08E-07
Th-228	3.80E-03	3.80E-03

Source: 2

Nuclide	Receptor 1	Total
K-40	4.52E-07	4.52E-07
Ra-226	1.67E-02	1.67E-02
Pb-210	2.79E-08	2.79E-08
Po-210	1.82E-08	1.82E-08
Th-232	2.60E-06	2.60E-06
Ra-228	8.33E-08	8.33E-08
Th-228	2.85E-03	2.85E-03

Source: 3

Nuclide	Receptor 1	Total
K-40	4.52E-07	4.52E-07
Ra-226	1.67E-02	1.67E-02
Pb-210	2.79E-08	2.79E-08
Po-210	1.82E-08	1.82E-08
Th-232	2.60E-06	2.60E-06
Ra-228	8.33E-08	8.33E-08
Th-228	2.85E-03	2.85E-03

Title : Default Case for RESRAD-BUILD

Input File : C:\RESRAD_Family\BUILD\4.0\UserFiles\OBM TEST 1.bld

Evaluation Time: 1.00000000 years

Source: 4

Nuclide	Receptor 1	Total
K-40	9.59E-04	9.59E-04
Ra-226	2.02E-02	2.02E-02
Pb-210	3.55E-08	3.55E-08
Po-210	1.86E-08	1.86E-08
Th-232	2.60E-06	2.60E-06
Ra-228	7.82E-05	7.82E-05
Th-228	2.89E-03	2.89E-03

Source: 5

Nuclide	Receptor 1	Total
K-40	9.59E-04	9.59E-04
Ra-226	2.02E-02	2.02E-02
Pb-210	3.55E-08	3.55E-08
Po-210	1.86E-08	1.86E-08
Th-232	2.60E-06	2.60E-06
Ra-228	7.82E-05	7.82E-05
Th-228	2.89E-03	2.89E-03
